# A Local Training Program to Increase Awareness of Emerging Extended Reality Technologies Among Health Care Professionals: Development Study

**DOI:** 10.2196/57361

**Published:** 2025-02-27

**Authors:** Charlotte Galvin, Jonathan Watt, Payal Ghatnekar, Nicholas Peres, Jacqueline Rees-Lee

**Affiliations:** 1 Torbay and South Devon NHS Foundation Trust, Torbay Hospital, Torquay, TQ2 7AA, United Kingdom, 01803 614567

**Keywords:** health care XR, extended reality in health care, XR, virtual reality in health care, VR, digital awareness training, digital deep dive, digital literacy, emerging health care technology, digital future, extended reality, virtual reality

## Abstract

**Background:**

Demands on health care services can greatly outweigh capacity. Multifactorial causative factors present great challenges, forcing the National Health Service (NHS) to increase efficiency and adaptivity. Concurrently, digital advancements are excelling and long-term plans for NHS sustainability are focusing on the use of technological interventions to benefit patients. As a result, integration of extended reality (XR) technology has become an important focus of health care research. However, models of how the digital literacy of health care workforces can be developed and how frontline staff can be actively involved in the design and development of creative digital interventions are lacking. Such programs are essential to allow the development and upscaling of digital innovation within the NHS for the benefit of the patients.

Such a program has been developed in the Digital Futures research lab at Torbay and South Devon NHS Foundation Trust, representing one of the first immersive digital technologies research spaces embedded within the NHS. A “Digital Deep Dive” training program has been developed, allowing local health care workers to recognize the possibilities of digital health care technologies and supporting them in the evolution of ideas for potential bespoke digital solutions appropriate to their own patient groups and care pathways.

**Objective:**

This paper aims to explain the development of this unique XR Deep Dive program and present the evaluation that informed future directions for its ongoing development.

**Methods:**

The Deep Dive sessions were designed according to relevant pedagogic principles, including experiential, active, and contextual learning theories. Voluntary pilot sessions were held for local clinical teams comprised of junior doctors, consultants, nurses, and allied health professionals. Self-selection sampling was used. Participants completed an anonymous postsession feedback form, which was used to conduct a service evaluation. Data were analyzed using descriptive statistics (quantitative) and thematic analysis (qualitative).

**Results:**

In total, 21 completed questionnaires were analyzed. Overall, the sessions were positively received: all participants reported increased awareness of the potential for digital health care innovation postsession and most found it useful and relevant to their clinical careers. Participants valued the sessions being grounded in a context relevant to local practice with opportunities to interact with the technology through the lens of use cases.

**Conclusions:**

We have developed a unique training initiative providing contextually relevant XR technology awareness training for health care professionals locally. Despite the growing pace of digital health care innovation, we recognized a knowledge gap in our local workforce regarding the potential of XR technologies within health care. We responded by developing a training program grounded in the concept of digital co-creation—working with staff and service users to develop bespoke solutions integrated within patient pathways. The results from this paper will help to inform future directions for developing digital awareness training in our trust and have implications for wider NHS digital literacy training.

## Introduction

We live in an era where demands on NHS services can outweigh capacity. This mismatch in capacity versus demand is increasing and causative factors are multifactorial, including an aging population, significant years of underfunding, a reducing and inadequate workforce, and the COVID-19 pandemic. To meet these challenges, health care services must become more adaptive and efficient, while maintaining a world-leading standard of patient and clinician experience, service quality, and clinical safety. It is also an era where technological and digital advancements are progressing at an unprecedented rate.

The 2019 government-commissioned Topol Review [1] made important recommendations to ensure the NHS becomes a world leader in digital technologies utilization for the benefit of patients, and the necessity to grow the digital literacy of the health care workforce was further accelerated by the COVID-19 pandemic [2]. In a more recent development, the 2023 NHS Long Term Workforce Plan [3] underscores the significance of digital competencies and integration as crucial components in equipping the workforce to meet prospective service demands.

Extended reality (XR)—an umbrella term encapsulating the spectrum of immersive technologies from simple augmented reality (AR) through to complete virtual reality (VR)—has become a key focus of cutting-edge health care research [4], with its benefits becoming clearer through use in as many as 97 UK health organizations and 119 distinct health care research projects in 2021 [5]. The comparison of XR-driven practices to traditional methods in medicine [6,7], surgery [8,9], rehabilitation [10,11], and clinical education [12,13] have become important research foci in recent years. Ultimately, the effectiveness of XR technologies in enhancing clinical skills and patient outcomes has been well demonstrated [6]. However, as important as these research projects are, they are insufficient if not accompanied by programs of digital training and education to reach the wider workforce.

A review of the literature has indicated that, while studies exploring the use of XR in a health care setting are numerous, real-world working models of health care workforce XR awareness training are lacking, with no applicable papers yielded from our search. Thus, despite the advancements in XR technologies within health care, there is a notable gap in the literature regarding the training of health care professionals to effectively integrate these tools into clinical practice for the benefit of patients. We propose that in order for XR technologies to be truly embedded in the NHS, within clinical care pathways and for the benefit of patients, they need to be understood and utilized by clinicians and health care professionals within the correct health care context. Although many digital technology companies are innovating in this space, direct access to and collaboration with clinicians and patients from the first stage of their innovation is lacking, meaning there is often a mismatch or lack of true co-design in what is being developed and what is actually required.

In 2020, this paper’s senior authors (JRL and NP) were profoundly aware of the lack of digital literacy within their local NHS health care workforce and the lack of successful fully integrated digital-clinical partnerships. Working together and alongside other experts to allow a true understanding of both the clinical and digital worlds, they set out to address this by conceptualizing and developing the unique “Digital Futures: Human Centred Digital Innovation” program [14], which was initially supported by funding from Health Education England. The idea was to allow a true understanding of both the clinical and digital worlds and develop innovations in the common ground between their areas of expertise. Thus, the conceptualization of a Digital Futures Research Lab built on an existing XR Lab, which had been in development at Torbay Hospital since 2016. The program represents one of the first immersive digital technologies research spaces embedded within the NHS to inform national insights into research and development of immersive digital technologies in health care.

The development of a “Digital Deep Dive” training program was one of the founding principles of the Digital Futures program. Its aim is to increase digital literacy and awareness in local clinical teams, supporting them to recognize the possibilities of digital health care technologies and evolve ideas for potential bespoke digital solutions appropriate to their own patient groups. The clinical user-led approach of joining digital experts and clinical experts was conceptualized to allow cross-fertilization of ideas and knowledge to support the creation of bespoke solutions within the patient pathways and represents a “bottom up” approach of educating staff groups in digital technology, which is now gaining national interest.

Through this paper, we aim to highlight how we have developed local XR Deep Dive Training Sessions as part of the Digital Futures Programme and evaluate the impact of pilot sessions we have delivered.

## Methods

### Design

The XR Deep Dive training sessions have been developed collaboratively between clinicians and digital experts at Torbay and South Devon Foundation Trust (TSDFT). The sessions were designed to be delivered to teams of health care professionals across the trust in the on-site TSDFT Digital Futures Research Lab. Since the authors consider cross-fertilization of digital and clinical expertise to be paramount in the development of digital interventions that are useful and usable in practice, the sessions were designed to be co-delivered by a clinician and a digital expert.

The Deep Dive learning strategy was originally conceptualized by a global learning design company in the early 2000s and has since been widely implemented across various industries to promote learning and process development within professional teams [15]. Core to the Deep Dive methodology is integration of key stakeholders, affording them the opportunity to experiment with new concepts and brainstorm how that concept could be adapted and successfully integrated into their own unique context [15]. This approach offers an ideal solution to the challenges of XR health care training we have previously described. Therefore, we have adapted the Deep Dive methodology to develop our local training program: we first introduce participants to the concept of XR, then we demonstrate its potential within health care, and finally we allow time, space, and support for teams to explore how the concept could be developed within the context of their own health care specialty for the direct benefit of local teams and patients.

To achieve this, we grounded our Digital Deep Dive session design in Experiential Learning theory [16]. A vital component of the deep dives is to showcase examples of embedded digital technologies in health care pathways across both our own trust and more widely, thus feeding the imaginations of the participants with the possibilities within the digital health care space by promoting hands-on experience and reflection [16]. In-session digital interaction was a key design priority, with time allocated to practical demonstrations and “digital playtime” allowing participants to trial the XR technology first hand. This also aligns with active learning theories and evidence that this type of digital interaction is a key component of achieving successful technology training [17]. The Digital Futures program has a “human first” approach to all its innovations, emphasizing how digital innovation can be utilized directly to improve patient care. In the Deep Dives, we therefore focus on technology in a humanistic sense—adopting this approach accentuates the personal, emotional, and psychological needs of the person in addition to their physical health needs, stressing the importance of treating each person as a unique individual, ensuring that care is patient-centred and that the health care experience is characterized by compassion, empathy, respect, and dignity [18]. We aimed to showcase how technology can be used to connect us with and value one another as fellow human beings, and so incorporated illustration of local use cases to provide context and authenticity. This design choice aligns with the goal of uniting concept with practice, which is central to contextual teaching and learning theory [19]. The informal learning environment was designed to encourage questions and discussion throughout, thereby supporting learners to develop a deeper understanding and explore different perspectives [20]. Time was also allotted at the end of the session for a mini focus group to further promote ideas for co-design and interdisciplinary collaboration of potential digital solutions. Sessions were designed to be delivered in a small group format (<10 participants), as this has been shown to foster better group collaboration, interaction, and discussion [20]. Finally, given the importance of posttraining follow-up to provide further support and ensure ongoing development [21], we considered how we would deliver postsession support as part of our program design—signposting to digital drop-in clinics to further improve targeted digital skills and share and refine ideas for future digital innovation was therefore promoted at the end of the Deep Dive sessions.

**Figure 1.**
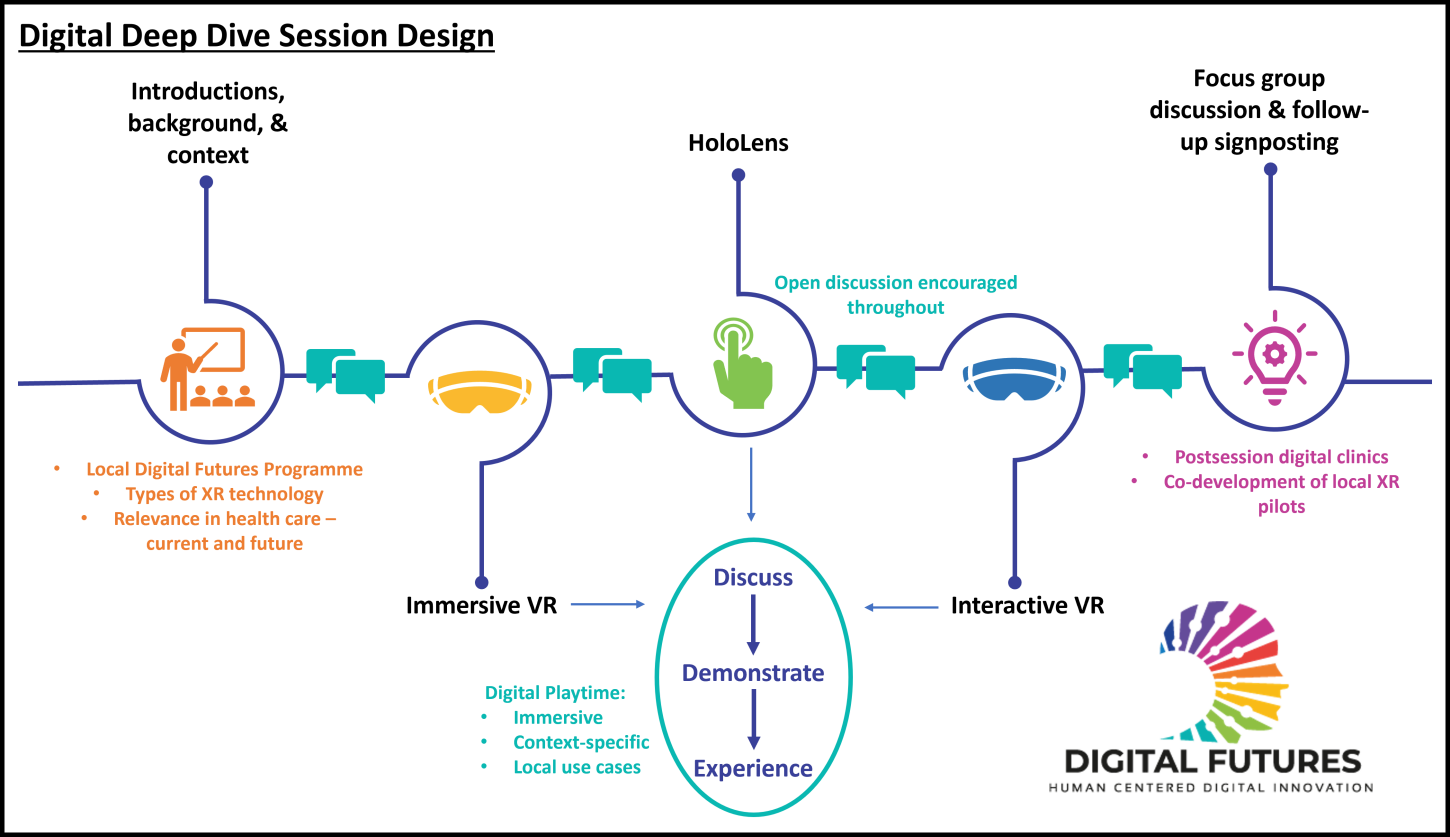
An outline of the design of the XR deep dive training sessions. VR: virtual reality; XR: extended reality.

**Figure 2.**
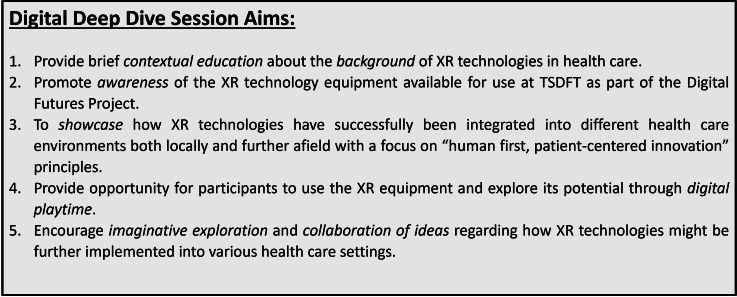
The aims of the XR deep dive training sessions. TSDFT: Torbay and South Devon Foundation Trust; XR: extended reality.

These design principles for the XR Deep Dive session are outlined in Figure 1, encapsulating the overarching aims of the training sessions, which are summarized in Figure 2.

Following the design phase, 8 voluntary pilot sessions were held between May 2022 and May 2023. Health care professionals—including resident doctors, consultants, nurses, occupational therapists, play specialists, and physiotherapists—from departments across TSDFT were invited between May 2022 and April 2023 via email and online trust advertising platforms to attend on a voluntary basis, therefore utilizing self-selection sampling [22]. Volunteers from all of these clinical groups attended sessions, with each session hosting between 3 and 8 participants to maintain the important small group sizes. Participants were invited to complete an anonymous postsession QR feedback form in Multimedia Appendix 1; by submitting this, participants consented for their anonymized comments to be included in this service evaluation. The Squire Guideline for Service Evaluation was used as a framework [23]. Free-text responses were evaluated by 2 authors (CG and PG) using thematic analysis, which is the accepted preferred method of interpreting qualitative data [24].

Each session was also observed by the senior author (JRL), who provided feedback on content and flow and suggested modifications. Using this feedback combined with the participant feedback, through an iterative process, the final content of the Digital Deep Dive sessions took shape.

### Ethical Considerations

In line with guidance provided by the Health Research Authority and compatible local Research and Development policies at TSDFT, a formal ethics application was not required for this service evaluation project. Participants were made aware through a formal statement on the feedback form that their anonymous responses may be used for evaluation purposes and may be included in future published work.

## Results

From a total of 8 sessions delivered to 35 participants, 21 completed questionnaires were received, with a mix of qualitative and quantitative responses (60% response rate). Quantitative responses were analyzed using descriptive statistics and free-text responses were thematically grouped and analyzed.

### Quantitative Data

Data were collected through a series of closed questions and 5-point Likert scales. Quantitative data were collected in 2 categories: presession experience and postsession feedback.

#### Presession Experience

Results are displayed in Table 1. All participants who took part in the XR Deep Dive sessions had little to no experience of using XR technology previously. Although just over half of participants were aware of XR being used in a health care context—either generally or specifically—the remainder had never heard of XR technologies being implemented in health care, and none had any personal involvement in using XR technologies in a health care context. Further, most participants had never heard of the Digital Futures Programme at TSDFT and knew nothing or very little about current use of XR technologies in our local health care services.

**Table 1.** Quantitative data (presession ideas).

Question and answer		Number of responses (N=21)	Percentage of total responses
**Before this session, what was your experience with virtual reality/augmented reality technologies?**			
	I had used these technologies a few times previously	11	52
	I had heard of these technologies but had never used them	9	43
	I had never heard of these technologies before	1	5
	I had lots of experience of using these technologies	0	0
**Before this session, how familiar were you with the use of digital technologies such as virtual reality/augmented reality in health care environments?**			
	I had never heard of these technologies being used in health care before	8	38
	I had heard of these technologies being utilized in health care but did not have much knowledge regarding how	7	33
	I had heard about specific projects involving these technologies in health care but have had no personal involvement	6	29
	I have personally been involved in projects utilizing these technologies in health care settings	0	0
**On a scale of 1‐5, how much did you previously know about the digital projects ongoing at Torbay and South Devon Foundation Trust?**			
	1 (absolutely nothing)	16	76
	2	2	10
	3	3	14
	4	0	0
	5 (expert)	0	0
**Had you previously heard of the Digital Futures Programme?**			
	Yes, and I knew what it was	1	4.76
	Yes, but I didn’t know what it was	1	4.76
	No	19	90.48

#### Postsession Feedback

Results are displayed in Table 2. All participants indicated that they had a better understanding of the Digital Futures Programme and ongoing XR projects within the trust after taking part in the session. Most participants felt that the session was both useful and relevant to their future clinical careers and reported feeling inspired or very inspired to utilize XR technologies in their own health care specialty. Most participants indicated that they felt to some degree more confident in operating the XR equipment after the session.

**Table 2.** Quantitative data (postsession feedback).

Question and answer		Number of responses (N=21)	Percentage of total responses
**Do you now have a better understanding of the Digital Futures Programme and the current digital projects ongoing in Torbay?**			
	Yes	21	100
	No	0	0
**On a scale of 1‐5, do you feel this session has inspired some ideas for how you might utilize digital technology in your chosen health care specialty?**			
	1 (not at all)	0	0
	2	0	0
	3	2	10
	4	7	33
	5 (completely)	12	57
**On a scale of 1‐5, how likely would you now be to get involved in a digital technologies in health care project in the future?**			
	1 (extremely unlikely)	0	0
	2	0	0
	3	4	19
	4	7	33
	5 (extremely likely)	10	48
**On a scale of 1‐5, how much more confident do you now feel in operating the virtual reality/HoloLens technologies compared to before the session?**			
	1 (not any more confident)	0	0
	2	0	0
	3	5	24
	4	13	62
	5 (entirely more confident)	3	14
**Do you think this session was useful to your future career?**			
	Yes	20	95
	No	0	0
	Unsure	1	5
**Do you think this session was relevant to your future career?**			
	Yes	20	95
	No	0	0
	Unsure	1	5

### Free-Text Data

**Figure 3.**
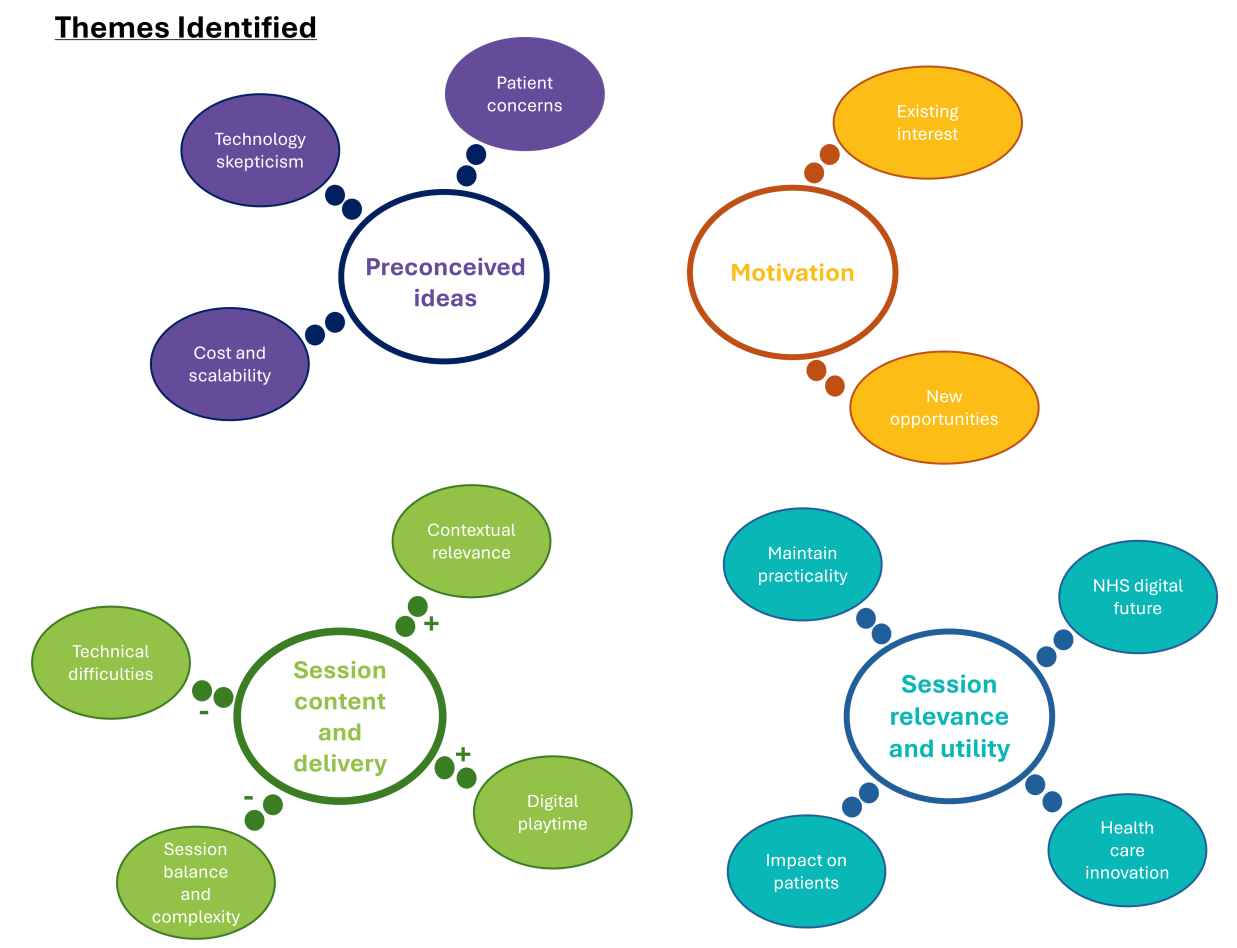
A visual representation of the themes identified from the free-text responses.

Free-text responses were collected in 4 main areas: presession ideas and motivation, session content and delivery; session relevance and utility; and postsession development. Following thematic analysis of the responses, key themes were identified in each of these areas. These themes are presented visually in Figure 3.

#### Presession Ideas and Motivation

Participants were asked 2 free-text questions in this area—the first related to presession ideas about technology use in health care and the second related to why the participant chose to get involved in a Deep Dive session.

Of the 21 respondents, 13 (62%) raised preconceived ideas about use of XR technology in health care. From these responses, 3 themes were identified: Patient Concerns, Technology Skepticism, and Cost and Scalability.

First, responses from 8 participants included concerns that highlight the preconceived ideas that technology would damage the patient-clinician relationship; technology use would lead to impersonal health care; and technology would present usability issues in certain patient groups, such as older patients. Together, these answers contribute to the dominant theme of Patient Concerns. Presented below are some direct quotes from the participants:

*I wondered how user-friendly the equipment might be, especially for older patients.*

[Participant 4]

*Worried about virtual technology replacing physical examination with patients.*

[Participant 19]

*Negative impact on the clinician-patient relationship—not very personal.*

[Participant 10]

Second, Technology Skepticism emerged as another preconceived idea. Participants expressed valid concerns about the relative infancy of XR technologies, particularly XR for health care, with some participant responses presented below:

*I know of such technology in the gaming world, but...I was skeptical about its uses in healthcare.*

[Participant 7]

*Technology and its use in healthcare are still very much in their infancy.*

[Participant 5]

The third theme that emerged from asking about preconceived ideas is that of Cost and Scalability. Four participants raised the concern that digital projects in health care may be unrealistic due to the costs involved, and its impact on availability and accessibility to the technologies. Some of the responses from the survey participants are presented below:

*Very costly so thought it would not be very achievable on a large scale.*

[Participant 8]

*Funding is likely to be the big barrier.*

[Participant 7]

Next, the motivation of respondents to participate in the Deep Dive sessions fell into 2 themes: Exploring an Existing Interest and Curiosity About New Opportunities. In response to the question about motivation for participating in the sessions, words such as “exciting,” “interesting,” and “unique” were used frequently.

An existing interest in digital technology was identified by 7 participants as motivation for their involvement in the training sessions. One participant stated:

*I am creative. I already know a bit about tech. I agree there is huge potential in using technology, specifically VR, to help people.*

[Participant 2]

Further, 13 participants talked about being curious about what they perceived to be a new and interesting area. Multiple participants alluded to technology being part of the future in health care and that it holds many opportunities for development. Some quotes from the participants are presented below:

*Interesting area of future development.*

[Participant 20]

*Wanted to hear more about what opportunity there was.*

[Participant 12]

#### Session Content and Delivery

Participants were asked to identify the best thing about the session and whether they had any improvement suggestions. To ensure future session improvements, a specific question was also asked about any difficulties participants experienced when using the digital technology.

Positive comments about the session content and delivery were grouped in 2 themes: Digital Playtime and Contextual Relevance.

When asked to identify the best thing about the session, participants overwhelmingly gave answers that can be categorized into the theme of Digital Playtime. The hands-on digital experience integral to the session design was met with substantive positivity, with 19 of 21 participants (90%) citing the opportunity to use the technology in the session as one of the best aspects. Some example survey responses are below:

*Fantastic to have hands on experience and understand more about how it all works.*

[Participant 11]

*Practical time with the headsets.*

[Participant 13]

Next, participants particularly valued the use of local case studies to illustrate real-life application and contextual relevance, with 6 participants commenting that integration of use cases into the session was one of its best aspects. One participant said it was:

*Brilliant to see the difference it’s already making in the trust and the collaboration and partnership working already going on.*

[Participant 11]

Participants were then asked about any specific technology difficulties experienced during the session and whether they had any improvement suggestions. Regarding technology difficulties, participants outlined 4 problems: connectivity issues (6 participants), motion sickness/nausea (2 participants), device fit issues (2 participants), and time to adjust (2 participants).

Eleven of 21 participants (52%) then made suggestions for session improvement. From the responses, 3 themes emerged: improvement of session balance, improvement of session complexity, and improvement of internet connectivity.

First, 8 participants gave answers that indicated better session balance would be welcomed. Integrating more digital playtime and less presentation time was frequently cited. Some participants suggested increasing the length of the session to allow for more digital playtime. One participant said:

*At times there was too much tech talk which meant less time spent using the actual equipment, I think this could be streamlined to make the best use of time in the session.*

[Participant 8]

Next, some responses suggested parts of the session were too complex and not pitched at the appropriate level. Participants highlighted that that there was “over-explanation of the technology” (Participant 1), “too much tech talk to start” (Participant 5), and that some parts of the session were “quite confusing” (Participant 4).

Finally, the quality of the internet connection was mentioned by 4 participants as an improvement suggestion, reinforcing that this was the main technology difficulty experienced during the sessions.

#### Session Relevance and Utility

Following the quantitative questions regarding session relevance and utility, participants were subsequently asked to explain their reasoning in a free-text question. Of the 21 participants, 20 (95%) thought the session was useful and relevant to their future clinical career—the single outlier was “unsure.” When asked to expand on their answers, participants gave responses in 4 themes: Digital Future of the NHS, Potential for Health Care Innovation, Impact on Patients, and Ensuring Ideas are Practical.

When considering the relevance/utility of the session, 11 of 21 participants (52%) commented on the Digital Future of the NHS and the need for the workforce to be knowledgeable and prepared:

*It will become more and more relevant over time.*

[Participant 8]

*Realise that tech is coming to the NHS and we need to be prepared to use it in our practice.*

[Participant 10]

*Tech is only going to become bigger in the next decade and clinicians need to catch up.*

[Participant 3]

Next, 5 participants gave answers that fall under the theme of Potential for Health Care Innovation, recognizing areas for digital integration such as development of virtual patient assessment systems and the interpretation of radiological imaging. The technology still being “in its early stages” (Participant 9), however, was also recognized.

Three participants wrote directly about the impact of technology on patients, which was considered from different angles:

*Still unsure whether this will benefit patients.*

[Participant 9]

*I can see how this type of thing can be used to benefits patients’ care in the future.*

[Participant 21]

Finally, 3 participants raised the point that that future innovations must be practical. Funding concerns were again mentioned as well as comments relating to the need to “work out what is realistic” (Participant 12) and the realization that some useful ideas “struggle in their execution” (Participant 2).

#### Postsession Development

To conclude, participants were asked for their suggestions on how the sessions should be followed up. From the 9 answers provided, 3 themes emerged: Clear Signposting, Focused Technology Support, and Exposure to Technology in Context.

The need for clearly signposted postsession support was raised by 3 participants, to allow ideas and interest generated in the session to be appropriately followed through. One participant talked about the benefit of having a “clear roadmap of steps from this workshop to generating ideas right through to fruition” (Participant 2).

Further, a need for focused technology support was identified by 4 participants, in order to provide more support to participants who had less experience with the technology itself or those who found adapting to the headsets more difficult. An example quote is included below:

*Would need more time and support if taking this forward as a project.*

[Participant 11]

Finally, 3 participants identified that they might benefit from the opportunity to have more exposure to the technology in context, perhaps with opportunities to trial it in clinical simulation or with real patients in the clinical environment.

## Discussion

An XR Deep Dive training program has been created for local health care professionals, which has been evaluated as being clinically relevant, successfully increasing local awareness of current digital innovation projects within health care. It is also potentially useful to future clinical practice. This is the first step in developing and enhancing digital literacy and innovation within our health care staff across our integrated care organization.

### Session Strengths

**Figure 4.**
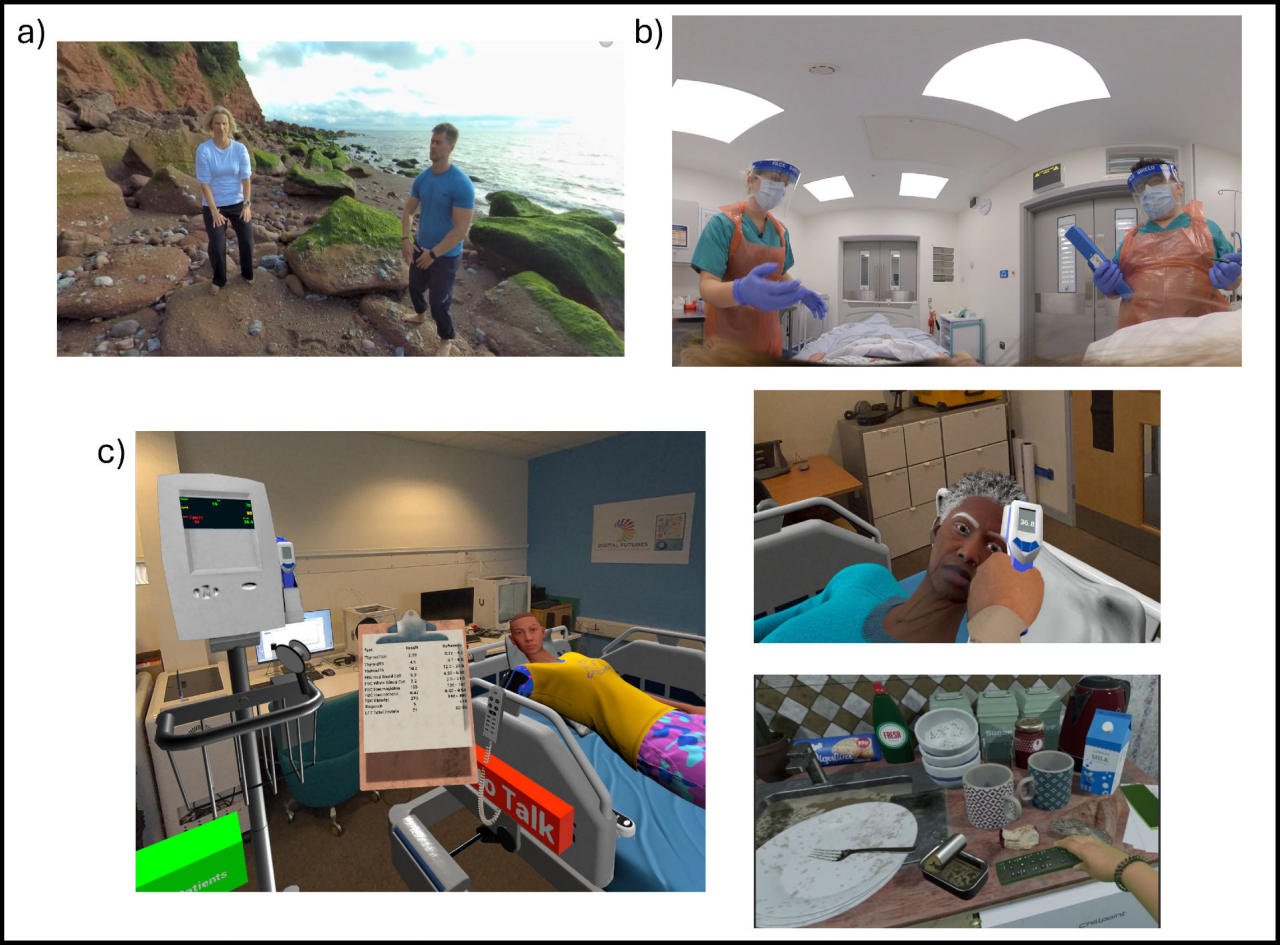
Examples of local XR use cases demonstrated during the XR deep dive training sessions: (A) immersive VR tai chi on the beach; (B) interactive VR empathy training; and (C) interactive HoloLens patient management training. VR: virtual reality; XR: extended reality.

Participants indicated that their presession experience of using XR technology was minimal to nonexistent. The integration of digital playtime and first-hand exposure to the technology were reported as being an overwhelming strength of the session. Participants were encouraged to reflect on these practical experiences and engage in collaborative group discussion about potential applications and developments in their own health care settings. This experiential learning is a key component of adult learning theory, where learning takes place in a context-specific cycle of experience, reflection, conceptualization, and experimentation [16]. To provide this all-important context, relevant local use cases of successful XR interventions formed the basis of the practical demonstrations, fueling participants’ imaginations of what is achievable within our own organization, thereby lifting the concept of XR integration from an abstract idea to a realistic possibility. For example, the following use cases (developed in-house) were explored (Figure 4):

1. Working with local clinical pain specialists, the Digital Futures team has been able to create a fully immersive tai chi on the beach VR experience (Figure 4A).
2. The successful integration of HoloLens technology to deliver immersive virtual clinics in the patient’s homes.
3. How XR technology has been used at TSDFT to develop and deliver interactive empathy (Figure 4B) and patient management training (Figure 4C).

This contextual relevance was another key strength in our survey results, supporting the mantra that “seeing is believing” where emerging technologies are concerned [25].

Significant cultural challenges exist to the widespread adoption of XR technologies across all industries, including feelings of apathy, distrust, confusion, and skepticism [25]. Such cultural barriers are reinforced through our survey, with more than half of respondents exhibiting negative preconceived ideas about the use of XR technology in health care across 3 themes: Patient Concerns, Technology Skepticism, and Cost and Scalability. We believe that such concerns must be addressed head-on by providing staff with the opportunity to experience the technology in action, with time and support to understand its qualities and limitations as well as openly discussing and addressing concerns [25]. After taking part in a Deep Dive session, many participants acknowledged the potential of XR technology for health care innovation and had developed an appreciation of what might be realistically achievable at a local level.

Our co-creation approach to developing digital solutions that are useful and usable in practice was fundamental to the design of the Digital Futures Deep Dive sessions and to addressing these concerns. Having access to a digital expert during the session enabled practical discussions focused on achievable digital goals. Emphasizing cross-fertilization of clinical and digital expertise allows participants to understand that our local Digital Futures Programme aims to produce co-developed, intelligently implemented, and practically driven bespoke patient-focused health care solutions [26], and that digital care transformations are taking place in a positive sphere of negotiation and meaningful dialogue with key stakeholders, rather than being forced upon them [27].

The success of our XR Deep Dive training sessions is encapsulated and demonstrated by a significant number of participants showing active postsession engagement and interest in becoming involved in the local Digital Futures Programme, bringing with them the seedlings of ideas that were sown in the initial XR Deep Dive session.

### Areas for Improvement

Our survey revealed that participants felt the balance and complexity of the session could be improved, with respondents requesting more hands-on time with the XR headsets and less presentation time, reinforcing that the strength of the session lies in its integration of practical digital experience. As a result of this feedback, we were able to perform a review of the session design after the first few deliveries and made some intermediate interventions, including increasing the session length from 90 to 120 minutes with more dedicated practical time, streamlining the session presentation, and simplifying the digital-focused background information. This resulted in improved feedback, with improvement comments under the themes of “Improvement of Session Balance” and “Improvement of Session Complexity” occurring far less frequently in the later pilot sessions.

Approximately one-quarter of respondents talked about connectivity issues when asked whether they experienced technology difficulties during the session. Resolution of connectivity issues subsequently became a theme for improvement. Such connectivity issues are unfortunately widespread in the NHS—a survey found that 58% of NHS staff had experienced Wi-Fi blind spots in their trust buildings, and two-thirds agreed that digital innovations in their team had been abandoned due to poor connectivity [26]. This is a limitation of NHS infrastructure and is not within the abilities of this paper’s authors to change. However, we recognize—like 98% of NHS staff—that Wi-Fi infrastructure and mobile connectivity are crucial to the future delivery of innovative health care [26] and will therefore continue to play our part in campaigning for improved connectivity as part of our local Digital Futures initiative.

### Principal Findings

The Digital Futures Lab is on-site in our NHS trust, and it is bespoke and evolving. It was built to develop and support the digital literacy of all health care staff in our trust. Our evaluation found that most participants came to our training session with no or little knowledge about the use of XR technologies in a health care context or local XR development projects. As expected, most participants had never heard of our new local Digital Futures Programme and were not aware of the investment and facilities available within our own organization recently. Without awareness of the opportunities available, clinical teams simply cannot drive digital innovation. This aligns with the findings of a 2023 survey that lack of digital knowledge and skills within health care teams was considered by three-quarters of surveyed NHS workers to be a significant barrier to innovation [26]. Furthermore, this emphasizes the Topol recognition that a culture of NHS digital innovation can only be achieved when coupled with a learning culture that supports frontline staff to explore new technologies and the opportunities they present for patient care [1].

An integral component to building such a culture is having a cohort of learners who are motivated to explore the opportunities presented by advancing digital technologies. An appetite to explore and embrace digital advancements to transform patient care has been identified among health care workers on a national scale [26]. Similarly, many participants in our survey acknowledged the upcoming digital age of the NHS, recognized digital technology as a tool for innovation, and cited their curiosity to learn about such innovation opportunities as motivation for seeking digital training.

Overall, in our local health care workforce, there exists a knowledge gap regarding XR potential and current local opportunities coupled with a strong desire to rectify this, indicating a clear need for the XR Deep Dive training session we have created. After taking part in our session, all respondents reported an increased awareness of local digital innovation and most felt inspired to get involved in future digital projects themselves, highlighting that our sessions have been successful in meeting their aims.

### Future Directions

Feedback from pilot sessions has supported the need for our new XR Deep Dive training sessions and has informed the refinement of the original session design as part of a quality improvement cycle. Intermediate interventions to address initial concerns regarding session balance and overcomplexity have already been successfully implemented, and there remains scope for further improvement. For example, future directions of the XR Deep Dive training program may involve a tiered approach to cater for participants of different starting abilities and experiences, potentially incorporating “beginner,” “intermediate,” and “advanced” training sessions, which can be accessed either in isolation or as a progressive series. Future evaluation of such an expansion of the training program would offer further insights into how we can successfully fulfill the NHS Long Term Workforce Plan of upskilling and training staff in our NHS trust to maximize digital technologies to improve health care delivery for the benefit of patients locally [3]. Future research will also inform us about the different technology behaviors of individuals and help us develop insights on how behavior change can be encouraged.

Digital transformation, and XR health care technologies in particular, are rapidly evolving and driving change. Maturation of hardware and software means content is becoming more sophisticated, user friendly, and seamlessly integrated into the real world [25]. Training programs—such as the one we have developed—will therefore also be required to evolve. Regular periodic reviews of the session content must be scheduled with updates as required, to ensure the training does not become outdated and irrelevant. Further, as use of technology in our local trust increases, the use cases demonstrated in the XR Deep Dive training sessions must also be reviewed to ensure they remain current and engaging. Showcasing use cases tailored to the participants’ own context will become easier as more local health care specialties adopt XR innovation.

As the training program grows, we must ensure its sustainability. This will involve the recruitment of local “clinical digital champions”—as identified in Topol [1]—to deliver peer-to-peer training, sharing their knowledge and unique experiences. Recruitment and training of digital experts must also be maintained—and increased proportionately—at the trust level. Ongoing funding must be secured in line with the program growth, which will require a funding strategy as part of the wider Digital Futures Programme in our trust. A robust and sustainable follow-up support model must be established to bridge the gap between this initial training session and adopting XR solutions in the clinical environment. Sparking the imagination of what is possible in the realm of local XR health care technology is trivial if participants do not subsequently have access to the technical support and expertise required to conduct trials within their own clinical spaces. We have already begun to tentatively explore a model of “Digital Clinics” for this purpose, but data from our survey emphasize how follow-up support must be focused, context-specific, and clearly signposted. Refining a sustainable follow-up model that meets these criteria is the next step in the development of this training program.

Finally, digital health care transformation is certainly not without its ethical challenges, including concerns around access, consent, inclusivity, privacy, and dignity [1,28]. As digital innovation training evolves, it must incorporate these ethical discussions and continue to tackle cultural barriers. Encouraging honest and open dialogue will be key to finding workable local solutions to ethical challenges and ensuring a true co-design culture is adopted. Our survey highlights staff concerns that XR technology will remove the personal aspect from patient-clinician relationships, thereby dehumanizing care. This concern is also recognized in the Topol review. Our local Digital Futures goal aligns with that of Topol: to focus on how digital technologies can enhance, rather than retract from, our human interactions. We are proud that our local digital projects prioritize the humanistic aspects of care and have built our training to showcase this. As digital innovation and the associated awareness training evolves, we must not lose sight of our core values.

### Limitations of This Paper

This paper explores a small, single-center pilot of a new local training intervention. Its findings are intended to inform future directions in our own trust and may not be generalizable to a wider context.

First, given the voluntary, self-selection sampling used to recruit participants to the Deep Dive pilot sessions, it is likely that our survey suffers from selection bias, capturing the views of staff who were already motivated to undertake the training in the first place. Given that a significant number of survey respondents talked about a prior interest in technology and a curiosity to explore new digital opportunities further as a reason to sign up to the pilot sessions, it is likely that our data do not capture the cohort of staff in our trust who are true digital skeptics. To obtain a wider spectrum of opinions, for future iterations of this pilot, we should aim to recruit staff members who do not have prior motivation for engaging in digital training sessions. This will provide insights into how we can effectively engage digital-skeptic staff to engage in the technology advancements being implemented both in our local trust and nationally within the health service.

Second, feedback was collected via an online feedback form accessed via a QR code at the end of the session. Not all session participants completed the feedback (60% response rate), possibly owing to the fact there was no physical form and they never got around to submitting it online. Concerns around nonresponse bias must therefore be considered when interpreting our findings. Obtaining feedback online is an established challenge [29]. To ensure a more complete representation of participant views in future, it may be preferable to supplement a feedback form with a recorded feedback focus group at the end of future sessions.

### Conclusion

Having identified a gap in real-world working models of health care workforce XR awareness and development training, we have designed and implemented XR Deep Dive training sessions for health care staff. This was one of the principle aims of our Digital Futures Programme. These sessions provide contextually relevant XR technology awareness training and are the first step in working toward the goal of nurturing digitally literate health care workforces who have the knowledge and skills to embrace transformative technology in the improvement of patient care, as per Topol [1]. Our session design draws on Experiential, Active, and Contextual Learning theories by showcasing local use cases of the technology in practice, prioritizing hands-on digital playtime and emphasizing the vital cross-fertilization of clinical and digital expertise in the co-creation of digital solutions that are useful and usable in practice. Data from the pilot sessions suggest that we have created a training session that is engaging as well as relevant and useful to future clinical practice. The results from this paper will help to inform future directions for developing digital awareness training in our trust.

## Supplementary material

10.2196/57361 Multimedia Appendix 1 The questionnaire survey used to collect participant feedback following pilot XR Deep Dive training sessions.

## Abbreviations

AR: augmented reality
NHS: National Health Service
TSDFT: Torbay and South Devon Foundation Trust
VR: virtual reality
XR: extended reality
