# Supplementary material for: A Local Training Program to Increase Awareness of Emerging Extended Reality Technologies Among Health Care Professionals: Development Study
Source: JMIR XR Spat Comput. 2025 Feb 27;2:e57361. doi: 10.2196/57361 (PMC12671294; doi:10.2196/57361)
Supplement: Multimedia Appendix 1 [file xr-v2-e57361-s001.pdf]

# Digital Deep Dive Pilot Feedback

Thank you for
taking part in a Digital Deep Dive Training session at Torbay and South Devon Foundation Trust and for completing this post-session feedback questionnaire.

These sessions
are being delivered locally as part of a pilot programme which we hope will inform future insights into how digital technologies in healthcare training sessions should be delivered. The digital lab and equipment used to deliver these sessions has received funding from Health Education England and the NHS Digital Futures Programme.

As part of our
research into digital technologies training, information gathered from the post-session feedback questionnaire you have completed may be used for evaluation and research purposes. This research may in the future be published. No information you have provided will be used in a manner which would allow identification of your individual responses. Please
note that due to the anonymous nature of the survey, **you will not be able to withdraw your response after it has been submitted.**

If you have any
further questions about this, they can be directed to the leads of the Digital Deep Dive project or the Co-Lead for the Digital Futures programme at TSDFT on the following contact details:

Dr Charlotte Galvin, Clinical
Teaching Fellow and Digital Deep Dives Project Lead, TSDFT – charlotte.galvin1@nhs.net

Jonathan Watt, Digital
Innovation Facilitator and Digital Deep Dives Project Lead, TSDFT – jonathan.watt4@nhs.net

Miss Jacqueline Rees-Lee,
Consultant Oncoplastic Breast Surgeon, Director of Medical Education and Local Digital Futures Co-Lead, TSDFT – jacqueline.rees-lee@nhs.net

* Indicates required question

1. Before this session, what was your experience with VR/AR technologies? *

*Mark only one oval.*

- ⬭ I had never heard of these technologies before
- ⬭ I had heard of these technologies but had never used them
- ⬭ I had used these technologies a few times previously
- ⬭ I had lots of experience of using these technologies

2. Before this session, how familiar were you with the use of digital technologies such as VR/AR in healthcare environments? *

*Mark only one oval.*

- ⬭ I had never heard of these technologies being used in healthcare before
- ⬭ I had heard of these technologies being utilised in healthcare but did not have much knowledge regarding how
- ⬭ I had heard about specific projects involving these technologies in healthcare but have had no personal involvement
- ⬭ I have personally been involved in projects utilising these technologies in healthcare settings

3. On a scale of 1-5, how much did you previously know about the digital projects ongoing at Torbay and South Devon Foundation Trust? *

*Mark only one oval.*

| | Absolutely nothing |
|---|---|
| 1 | ◯ |
| 2 | ◯ |
| 3 | ◯ |
| 4 | ◯ |
| 5 | ◯ |
| | Expert |

4. Had you previously heard of the Digital Futures Programme? *

*Mark only one oval.*

◯ No
◯ Yes, but I didn't know what it was
◯ Yes, and I knew what it was

5. Before this session did you have any pre-conceived ideas, thoughts, hesitancies or concerns about the use of digital technologies in healthcare settings? *

_____________________________________________
_____________________________________________
_____________________________________________
_____________________________________________
_____________________________________________

6. What made you want to get involved in the Digital Deep Dive Training sessions?

_______________________________________________

_______________________________________________

_______________________________________________

_______________________________________________

_______________________________________________

7. Do you now have a better understanding of the Digital Futures Programme and the current digital projects ongoing in Torbay and South Devon Trust? *

*Mark only one oval.*

- ( ) Yes
- ( ) No
- ( ) Unsure

8. On a scale of 1-5, do you feel this session has inspired some ideas for how you might utilise digital technology in your chosen healthcare specialty? *

*Mark only one oval.*

Not at all

1 ( )

2 ( )

3 ( )

4 ( )

5 ( )

Completely

9. On a scale of 1-5, how likely would you now be to get involved in a digital technologies in healthcare project in the future? *

*Mark only one oval.*

Extremely unlikely

1 ◯

2 ◯

3 ◯

4 ◯

5 ◯

Extremely likely

10. On a scale of 1-5, how much more confident do you now feel in operating the VR/HoloLens technologies compared to before the session? *

*Mark only one oval.*

I do not feel any more confident at operating these technologies

1 ⬭

2 ⬭

3 ⬭

4 ⬭

5 ⬭

I feel entirely more confident at operating these technologies

11. Did you experience any difficulties in using the digital technologies? Please explain what these were if so

12. Do you think this session was useful to your future career? *

*Mark only one oval.*

⬭ Yes

⬭ No

⬭ Unsure

13. Do you think this session was relevant to your future career? *

*Mark only one oval.*

- ( ) Yes
- ( ) No
- ( ) Unsure

14. Can you explain your answers?

_______________________________________________
_______________________________________________
_______________________________________________
_______________________________________________
_______________________________________________

15. What was the best thing about this session? *

_______________________________________________
_______________________________________________
_______________________________________________
_______________________________________________
_______________________________________________

16. Is there anything about the session that could be improved?

_______________________________________________
_______________________________________________
_______________________________________________
_______________________________________________
_______________________________________________
